# The level of education is associated with an anxiety-depressive state among men and women – findings from France during the first quarter of the COVID-19 pandemic

**DOI:** 10.1186/s12889-023-16280-9

**Published:** 2023-07-21

**Authors:** Camille Joannès, Niamh M. Redmond, Michelle Kelly-Irving, Josephine Klinkenberg, Cassandra Guillemot, Florence Sordes, Cyrille Delpierre, Lola Neufcourt, On behalf of the EPIDEMIC consortium, Basson Jean-Charles, Beltran Grégory, Boulaghaf Laurence, Cave Alizé, Cipriani Enzo, Coeurdevey Eleonore, Croiset Aurélie, Delpierre Cyrille, Faya-Robles Alfonsina, Kelly-Irving Michelle, Maurel Marine, Nicaise Sarah, Soulier Alexandra, Srocynski Meryl

**Affiliations:** 1 Centre d’épidémiologie et de recherche en santé des populations (CERPOP) - UMR1295, Equity Research Team, Inserm, Université Toulouse III Paul Sabatier, Toulouse, France; 2 Interdisciplinary Federal Research Institute On Health and Society (IFERISS-Fed 4241), Université Toulouse III Paul Sabatier, Toulouse, France; 3 Centre d’étude et de recherche en psychopathologie et psychologie de la santé (CERPPS) - EA7411, Université Toulouse II, Toulouse, France

**Keywords:** Mental health, Education, Social health inequalities, SARS-CoV-2

## Abstract

**Context:**

It is widely recognised that the COVID-19 pandemic has negatively impacted individuals’ mental health. However, little emphasis has been put on the possible influence of socio-economic factors in the relationship. In the context of the COVID-19 pandemic, our objectives were (i) to assess the relationship between education level and mental health in French adults, and (ii) to study the influence of the economic, social, health and the COVID-19-related factors in men and women respectively.

**Method:**

Data are from 32,581 individuals representative of the French population who responded to the weekly survey “Baromètre COVID-19” between April 7^th^ and May 31^st^ 2020. Education level was self-reported (university degree, high school qualification, vocational certificate/qualification, no diploma). Anxiety-depressive state was derived from four items related to the frequency of occurrence of depressive and anxiety symptoms, and summarized in an overall validated anxiety-depressive score. Multivariate linear regression analyses were carried out with nested adjustments of variables related to economic, social, health and COVID-19 contexts to assess the relationship between education and anxiety-depressive state.

**Results:**

In total, 45% of individuals reported symptoms of anxiety-depressive state (53% in women versus 36% in men). Among men, those with a vocational certificate/qualification and those with no diploma had a greater risk of having a higher anxiety-depressive state compared to those with a university degree (β_Vocational certificate/qualification_ = 0.16 [0.04; 0.27]; β_No diploma_ = 0.75 [0.43; 1.07]) while among women, the risk of anxiety-depressive state increased as education level decreased (β_Baccalaureate_ = 0.37 [0.25; 0.49]; β_Vocational certificate/qualification_ = 0.41 [0.28; 0.54]; β_No diploma_ = 0.8 [0.49; 1.12]). For both men and women, economic, health, and COVID-19 factors partly attenuate these associations while social factors marginally modified the relationship. After accounting for confounders and intermediate variables, the absence of a diploma remained associated with anxiety-depressive state among men, while the whole educational gradient of anxiety-depressive state persisted among women.

**Conclusion:**

In France, at the end of the first wave of COVID-19, individuals with a lower level of education had a higher risk of anxiety-depressive state. This association was more pronounced for women, highlighting a process of social inequality in health possibly related to gender. This should be considered in future prevention and public health interventions.

## Introduction

The COVID-19 pandemic and the subsequent economic downturn have had a negative impact on the mental health of individuals [1, 2]. Good mental health is not just the absence of mental illness or disorder but has been more recently defined by well-being and the ability to enjoy life and adapt to the challenges we face [3]. The WHO reported that social isolation resulting from lockdown measures has led to an increasing incidence of anxiety and sleep disorders, suicidal ideation, and substance abuse [4]. Indeed, the COVID-19 pandemic is recognised as having had direct and indirect psychological and social effects that can affect the mental health of individuals, both at the time of the pandemic and afterwards [5]. However, most of the published literature focusses on specific populations: children or adolescents [6], students [7], healthcare workers [8] and those already affected by mental disorders [9]. Only a few reviews have looked at mental health in the general population [10, 11], with little emphasis put on the possible role of socio-economic position in relation to mental health in the context of the COVID-19 pandemic.

In France, where the first "lockdown" was relatively restrictive compared to other European countries [12], the CoviPrev survey investigated behaviour changes associated with the pandemic (safety measures, lockdowns, alcohol and tobacco consumption, diet and physical activity) and mental health (well-being, disorders), and found that job instability was associated with an increase in these disorders, particularly among socio-economically vulnerable adults [13]. However, to our knowledge, there is limited evidence on how other socio-economic factors, such as educational level, commonly used as an indicator of socio-economic position [14], have impacted the mental health of individuals in the context of the COVID-19 pandemic, resulting in social health inequalities in France.

A lower education level has been found to be associated with poorer mental health, and with a greater risk of developing mental health disorders [15]. Differences between men and women have been reported in mental health disorders like anxiety, depression [16]. In general, women are more often represented in the internalizing spectrum, including depressive, anxiety, eating and somatoform disorders, while men are more likely to have externalizing disorders such as substance abuse, impulse control and antisocial personality disorders [17]. The origins of these differences could be explained by socio-economic factors, since adults, and especially women [18], with low levels of education are more exposed to stressful environments, and are more likely to be affected by difficult or changing economic and material living conditions [19]. Furthermore, cultural and social influences differently affecting men and women such as marital status, family structure or gender-related socialization, could also explain these differences [20], as well as gender differences in behaviours affecting health system use, whether in the use of care, prevention or through gender biases in response to medical or healthcare questionnaires [21]. Thus, economic, social, and health factors could explain the influence of education on mental health, possibly in different ways in men and women. Given that social inequalities in health were exacerbated by the COVID-19 pandemic [22], and that women were more affected by psychological distress than men [11, 23], this raises the question of whether there are factors related to this pandemic context that can also potentially explain the association between education and mental health in men and women.

In the context of the COVID-19 pandemic, our objectives were (i) to assess the relationship between the level of education and mental health among men and women in France, and (ii) to study the influence of the economic, social, health contexts and COVID-19 context factors as potential mediating factors in this association. Our work focused on anxiety-depressive state as a mental health outcome, which is a symptom prevalent in both men and women in France and is associated with other mental health conditions [24]. We hypothesized (i) that a lower level of education was associated with a higher risk of anxiety-depressive state and that this differed for men and women; (ii) that this relationship could be at least partly explained by economic, social, health contexts and/or by factors related to the COVID-19 pandemic context in both men and women.

## Methods

### Study design and participants

The “Baromètre COVID-19” is a weekly national survey that was conducted during spring 2020 and which aimed to inform the French response to the COVID-19 pandemic, with self-reported data freely available at: https://www.data.gouv.fr/fr/datasets/datacovid-barometre-covid-19/#/resources/. This resulted from a partnership between IPSOS, AGALIO and other sponsors. Each week, a web-based survey was administered by the IPSOS polling institute to a sample of 5000 people representative of the French population, aged 18 and over (pseudo-panel), established by the quota method (sex, age, occupation, region and urban area). For this study, 35,001 participants were surveyed between April 7^th^ and May 31^st^ 2020, which corresponds to the fourth to eighth week of the first lockdown across the whole of France. Within this sample, 32,581 individuals provided complete data for our analyses.

### Outcome

Anxiety-depressive state was estimated using a composite score based on participants’ self-report of the frequency of occurrence of four items: Feeling sad, depressed, or hopeless; Feeling nervous, anxious, or tense; Being unable to stop worrying or control worrying; Having little interest or pleasure in doing things (Never = 0, Hardly ever = 1, Sometimes = 2, More than half the days = 3, More than one day = 4). These items were based on the validated Patient Health Questionnaire-4 (PHQ-4) [25], a simplified screening tool for anxiety and depression, and were translated into French. The sum of these four items resulted in an overall anxiety-depressive score ranging from 0 to 16 (a higher score representing more frequent symptoms). Cronbach's alpha [26] was calculated (α = 0.86) to ensure reliability of the score. We dichotomized this score (“No anxiety-depressive state/Anxiety-depressive state”) according to the cut-off of 3 [25]. This cut-off is based on the receiver operating characteristic (ROC) analyses conducted in previous validation studies [27, 28]. We used this binary variable in descriptive and bivariate analyses and the continuous score in multivariate analyses.

### Main exposure

Education was our main exposure of interest. Based on participants’ self-report, the education variable was divided into four categories: university degree, baccalaureate (or high school qualification), vocational certificate or qualification, no diploma [29].

### Confounders

The confounding variables available to measure were age (10-year increments, from 18 to 65 years and over), and the size of the residential agglomeration pre-categorized by the survey (rural; 2 000–19 999 inhabitants; 20 000–99 999 inhabitants; more than 100 000 inhabitants; Paris (> 1 million)).

### Intermediates variables

Economic factors were measured using occupation, grouped according to the French classification system (managers; farmers; self-employed; intermediate professions; employees; manual workers; retired persons; inactive/unemployed [30]) and overcrowding in the household (the number of people per room [14]).

Social factors comprised marital status (single; cohabiting; married; separated/divorced/widowed; civil partnership) and the presence of dependent minors in the household (none; one; two or more).

Health factors [31, 32] were represented by self-reported comorbidities with regard to the following: diabetes, cancers, respiratory diseases, chronic renal failure on dialysis, chronic liver disease, hypertension or heart disease, immune diseases and immunosuppressive treatment (none; one; two or more). Self-reported body mass index (BMI) was also considered and was coded into four groups according to the WHO cut-off points (< 18 kg/m^2^; [18–25[ kg/m^2^, [25–30[ kg/m^2^ and > 30 kg/m^2^).

Finally, the COVID-19 context factors included self-reported SARS-CoV-2 infection status (no infection; infection diagnosed by test or medical examination; suspected infection), data collection waves (during lockdown and before the "lockdown being lifted" announcement^1^; during lockdown and after the "lockdown being lifted" announcement; during the "lockdown lifted" period), frequency of social contact (none; low; medium; high), occupational status during lockdown (outside the home; at home) and perceived severity of the pandemic (0–10 scale).

**Fig. 1:**
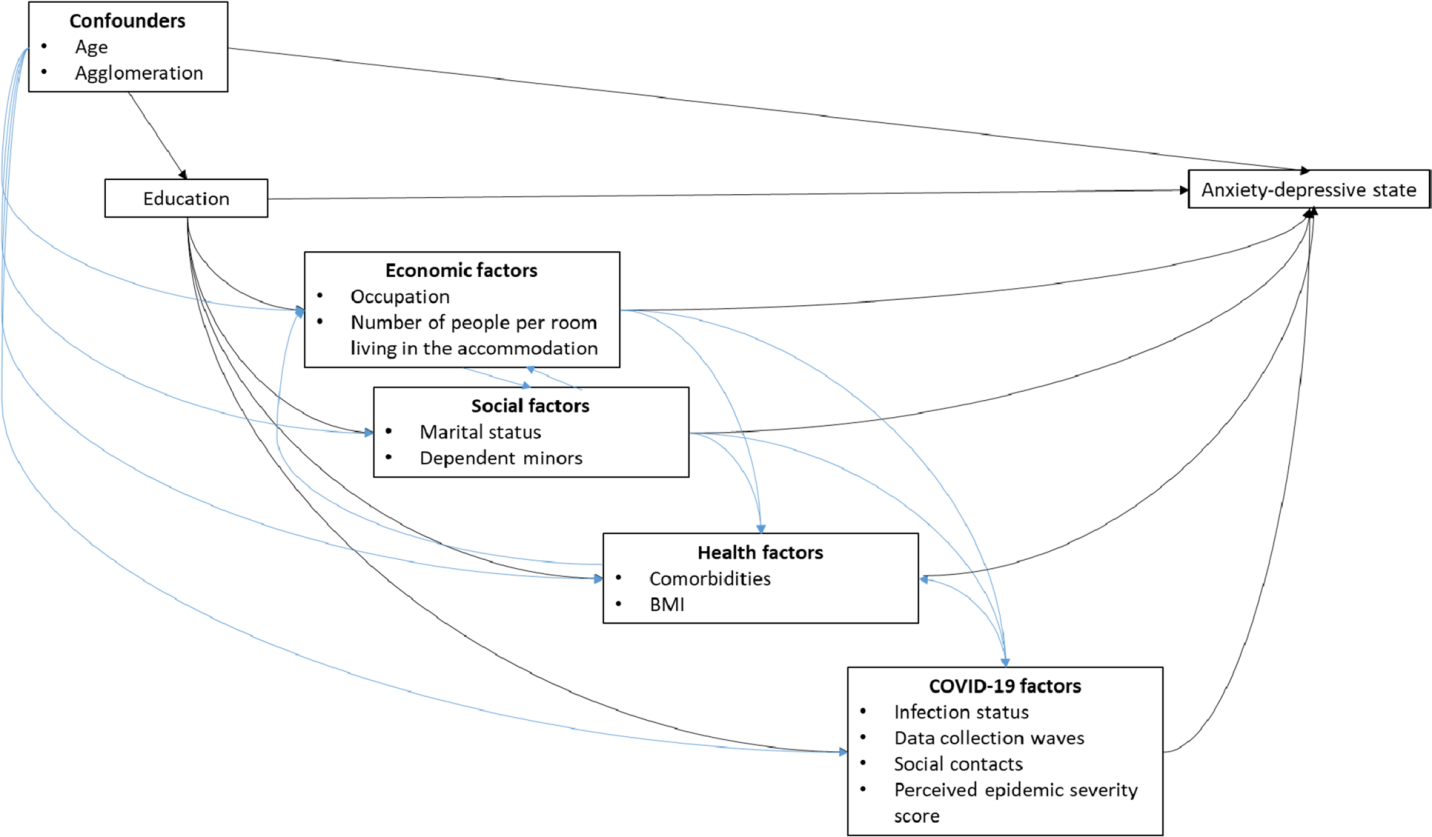
Causal diagram of the study

Figure 1 represents the theoretical causal diagram of the study.

### Statistical analysis

Comparisons of participants' characteristics by anxiety-depressive state were made using the Pearson Chi^2^ test or the Wilcoxon signed-rank test on the binary outcome. The relationship between education level and anxiety-depressive score, and the influence of each of the intermediate factors on this relationship were investigated by nested linear regression models, stratified by sex:

- Model 1: Anxiety-depressive score ~ Education level + confounders.
  - ◦ Model 1A: Model 1 + Economic factors (occupation + number of people per room living in the accommodation)
  - ◦ Model 1B: Model 1 + Social factors (marital status + dependent minors)
  - ◦ Model 1C: Model 1 + Health factors (comorbidities + BMI)
- Model 2: Model 1 + Economic factors + Social factors + Health factors
- Model 3: Full model: Model 2 + COVID-19 factors (infection status + response period + social contacts + perceived epidemic severity score)

In order to assess the contribution of the different factors in explaining the association observed in Model 1, we estimated the change in effect size with subsequent adjustments between the estimated regression coefficients for the education variable on anxiety-depressive score. We defined a final model (Model 3) including all the intermediate variables with the COVID-19 factors to assess the residual effect of education on anxiety-depressive score.

The analyses were performed using STATA v17 [33].

## Results

### Characteristics of the sample

The descriptive characteristics of the male and female subsamples are presented in Table 1. Among 32,581 complete case participants, 55% were women and 45% of individuals reported symptoms of anxiety-depressive state. About half of the participants were over 50 years old, lived in densely populated cities and had a university degree.

**Table 1:** Characteristics of the study sample “Baromètre Covid-19”, stratified by sex (*n* = 32,581)

		**SEX**			
Variable	Levels	Men *n*(%)	Women *n*(%)	Total *n*(%)	*p*-value
**Anxiety-depressive state**	No	9,499 (64%)	8,311 (47%)	17,810 (55%)	< 0.001
	Yes	5,323 (36%)	9,448 (53%)	14,771 (45%)	
**Education level**	University degree	7,660 (52%)	9,832 (55%)	17,492 (54%)	< 0.001
	Baccalaureate	3,068 (21%)	4,048 (23%)	7,116 (22%)	
	Vocational certificate/qualification	3,778 (25%)	3,477 (20%)	7,255 (22%)	
	No diploma	316 (2%)	402 (2%)	718 (2%)	
**Age**	18y to 29y	1,693 (11%)	3,006 (17%)	4,699 (14%)	< 0.001
	30y to 39y	2,126 (14%)	3,577 (20%)	5,703 (18%)	
	40y to à 49y	2,577 (17%)	3,332 (19%)	5,909 (18%)	
	50y to 59y	2,658 (18%)	2,766 (16%)	5,424 (17%)	
	60y to 64y	1,610 (11%)	1,600 (9%)	3,210 (10%)	
	65y and over	4,158 (28%)	3,478 (20%)	7,636 (23%)	
**Population density**	Rural	2,999 (20%)	3,734 (21%)	6,733 (21%)	0.334
	2,000–19,999]	2,646 (18%)	3,064 (17%)	5,710 (18%)	
	20,000–99,999]	2,122 (14%)	2,493 (14%)	4,615 (14%)	
	> 100,000	4,825 (33%)	5,798 (33%)	10,623 (33%)	
	Paris	2,230 (15%)	2,670 (15%)	4,900 (15%)	
**Occupation**	Managers	1,909 (13%)	1,660 (9%)	3,569 (11%)	
	Farmers	73 (0%)	72 (0%)	145 (0%)	< 0.001
	Self-employed	567 (4%)	518 (3%)	1,085 (3%)	
	Intermediate professions	2,330 (16%)	3,010 (17%)	5,340 (16%)	
	Employees	1,784 (12%)	4,528 (26%)	6,312 (19%)	
	Manual workers	1,884 (13%)	757 (4%)	2,641 (8%)	
	Retired	5,189 (35%)	4,386 (25%)	9,575 (29%)	
	Inactive/unemployed	1,086 (7%)	2,828 (16%)	3,914 (12%)	
**Number of people per room**	< 1.5	14,590 (98%)	17,465 (98%)	32,055 (98%)	0.520
	≥ 1.5	232 (2%)	294 (2%)	526 (2%)	
**Marital status**	Single	2,917 (20%)	3,988 (22%)	6,905 (21%)	< 0.001
	Unmarried union	1,883 (13%)	2,600 (15%)	4,483 (14%)	
	Married	7,497 (51%)	6,907 (39%)	14,404 (44%)	
	Separated/divorced/widowed	1,545 (10%)	2,962 (17%)	4,507 (14%)	
	Civil partnership	980 (7%)	1,302 (7%)	2,282 (7%)	
**Dependent minors**	None	10,571 (71%)	11,455 (65%)	22,026 (68%)	< 0.001
	One	2,029 (14%)	3,013 (17%)	5,042 (15%)	
	Two or more	2,222 (15%)	3,291 (19%)	5,513 (17%)	
**Comorbidities**	None	10,080 (68%)	13,634 (77%)	23,714 (73%)	< 0.001
	One	3,292 (22%)	3,033 (17%)	6,325 (19%)	
	Two or more	1,450 (10%)	1,092 (6%)	2,542 (8%)	
**BMI**^1^	Normal weight ([18–25[ kg/m^2^)	6,222 (42%)	9,310 (52%)	15,532 (48%)	< 0.001
	Underweight (< 18 kg/m^2^)	253 (2%)	1,030 (6%)	1,283 (4%)	
	Overweight ([25–30[ kg/m^2^)	5,696 (38%)	4,481 (25%)	10,177 (31%)	
	Obesity (> 30 kg/m^2^)	2,651 (18%)	2,938 (17%)	5,589 (17%)	
**SARS-CoV-2 infection**	No infection	13,140 (89%)	15,750 (89%)	28,890 (89%)	< 0.001
	Diagnosed by test or medical examination	496 (3%)	742 (4%)	1,238 (4%)	
	Suspected	1,186 (8%)	1,267 (7%)	2,453 (8%)	
**Data collection waves**	Before the "lockdown being lifted" announcement	2,196 (15%)	2,466 (14%)	4,662 (14%)	0.030
	After the "lockdown being lifted" announcement	8,393 (57%)	10,264 (58%)	18,657 (57%)	
	During the "lockdown lifted" period	4,233 (29%)	5,029 (28%)	9,262 (28%)	
**Frequency of social contacts**	None	7,659 (52%)	9,518 (54%)	17,177 (53%)	0.001
	Low (< 2)	3,052 (21%)	3,541 (20%)	6,593 (20%)	
	Medium ([3, 4])	2,080 (14%)	2,279 (13%)	4,359 (13%)	
	High (5–200]	2,031 (14%)	2,421 (14%)	4,452 (14%)	
**Occupational status during lockdown**	At home	11,749 (79%)	14,638 (82%)	26,387 (81%)	< 0.001
	Out of home	3,073 (21%)	3,121 (18%)	6,194 (19%)	
**Perceived severity of the pandemic (mean [p25-p75])**		7.77 [7; 9]	8.14 [7; 10]	7.97 [7; 9]	< 0.001
	**Total**	14,822 (100%)	17,759 (100%)	32,581 (100%)	

^1^*BMI* Body mass index

Women were more likely to report an anxiety-depressive state than men (53% versus 36%) and were more likely to have a university degree, a baccalaureate or no degree compared to men. In addition, compared to men, women were more likely to be in intermediate occupations, employed or inactive, single, separated/divorced/widowed or cohabiting, living with a dependent minor in the household. They were less likely to suffer from comorbidity and obesity, but had a higher risk of being diagnosed with Sars-Cov-2 by a medical test or examination. Conversely, men were more likely to be managers, farmers, self-employed, manual workers or retired, married, to work outside the home during the lockdown, to have more social contacts outside the household and perceive the severity of the pandemic as lower than that of women.

### Factors associated with anxiety-depressive state in men and women

Bivariate analyses by anxiety-depressive state are presented for men in Table 2A and for women in Table 2B. With regard to education level, men with an anxiety-depressive state tended to have a university degree or no educational qualifications, while women with an anxiety-depressive state tended to have a baccalaureate or no educational qualifications. In addition, for both men and women, those with anxiety-depressive state tended to be under 50 years of age, to live in populated areas and in overcrowded housing, to be single or unmarried, to live with dependent minors, to have one or more comorbidities, to have contracted COVID-19 (confirmed by test or suspected), to have had social contacts during the lockdown and to have considered the pandemic as severe or very severe compared to those without anxiety-depressive state. Among men, those who reported anxiety-depressive state tended to be employed or unemployed but not retired, and to have worked outside their home during the lockdown, compared to those without anxiety-depressive state. Conversely, among women, anxiety-depressive state was more likely to be found among those who were employed, manual workers or unemployed, compared to those without anxiety-depressive state.

**Table 2:** Anxiety-depressive state by confounding and intermediate variables for men and women (*n* = 32,581)

		**A. Men**				**B. Women**			
		**Anxiety-depressive state**				**Anxiety-depressive state**			
Variable	Levels	No *n*(%)	Yes *n*(%)	Total *n*(%)	*p*-value	No *n*(%)	Yes *n*(%)	Total *n*(%)	*p*-value
**Education level**	University degree	4,833 (51%)	2,827 (53%)	7,66 (52%)	< 0.001	4,683 (56%)	5,149 (55%)	9,832 (55%)	< 0.001
	Baccalaureate	1,982 (21%)	1,086 (20%)	3,068 (21%)		1,79 (22%)	2,258 (24%)	4,048 (23%)	
	Vocational certificate/qualification	2,519 (27%)	1,259 (24%)	3,778 (25%)		1,668 (20%)	1,809 (19%)	3,477 (20%)	
	No diploma	165 (2%)	151 (3%)	316 (2%)		170 (2%)	232 (2%)	402 (2%)	
**Age**	18y to 29y	804 (8%)	889 (17%)	1,693 (11%)	< 0.001	1,042 (13%)	1,964 (21%)	3,006 (17%)	< 0.001
	30y to 39y	1,152 (12%)	974 (18%)	2,126 (14%)		1,516 (18%)	2,061 (22%)	3,577 (20%)	
	40y to à 49y	1,487 (16%)	1,090 (20%)	2,577 (17%)		1,539 (19%)	1,793 (19%)	3,332 (19%)	
	50y to 59y	1,718 (18%)	940 (18%)	2,658 (18%)		1,375 (17%)	1,391 (15%)	2,766 (16%)	
	60y to 64y	1,142 (12%)	468 (9%)	1,610 (11%)		859 (10%)	741 (8%)	1,600 (9%)	
	65y and over	3,196 (34%)	962 (18%)	4,158 (28%)		1,980 (24%)	1,498 (16%)	3,478 (20%)	
**Population density**	Rural	1,989 (21%)	1,010 (19%)	2,999 (20%)	< 0.001	1,868 (22%)	1,866 (20%)	3,734 (21%)	< 0.001
	2,000–19,999]	1,792 (19%)	854 (16%)	2,646 (18%)		1,438 (17%)	1,626 (17%)	3,064 (17%)	
	20,000–99,999]	1,358 (14%)	764 (14%)	2,122 (14%)		1,193 (14%)	1,300 (14%)	2,493 (14%)	
	> 100,000	3,023 (32%)	1,802 (34%)	4,825 (33%)		2,542 (31%)	3,256 (34%)	5,798 (33%)	
	Paris	1,337 (14%)	893 (17%)	2,230 (15%)		1,270 (15%)	1,400 (15%)	2,670 (15%)	
**Occupation**	Managers	1,152 (12%)	757 (14%)	1,909 (13%)	< 0.001	780 (9%)	880 (9%)	1,66 (9%)	< 0.001
	Farmers	30 (0%)	43 (1%)	73 (0%)		31 (0%)	41 (0%)	72 (0%)	
	Self-employed	308 (3%)	259 (5%)	567 (4%)		247 (3%)	271 (3%)	518 (3%)	
	Intermediate professions	1,428 (15%)	902 (17%)	2,33 (16%)		1,457 (18%)	1,553 (16%)	3,010 (17%)	
	Employees	958 (10%)	826 (16%)	1,784 (12%)		1,893 (23%)	2,635 (28%)	4,528 (26%)	
	Manual workers	1,099 (12%)	785 (15%)	1,884 (13%)		313 (4%)	444 (5%)	757 (4%)	
	Retired	3,969 (42%)	1,220 (23%)	5,189 (35%)		2,487 (30%)	1,899 (20%)	4,386 (25%)	
	Inactive/unemployed	555 (6%)	531 (10%)	1,086 (7%)		1,103 (13%)	1,725 (18%)	2,828 (16%)	
**Number of people per room**	< 1.5	9,398 (99%)	5,192 (98%)	14,59 (98%)	< 0.001	8,2 (99%)	9,265 (98%)	17,465 (98%)	0.002
	≥ 1.5	101 (1%)	131 (2%)	232 (2%)		111 (1%)	183 (2%)	294 (2%)	
**Marital status**	Single	1,577 (17%)	1,340 (25%)	2,917 (20%)	< 0.001	1,674 (20%)	2,314 (24%)	3,988 (22%)	< 0.001
	Unmarried union	1,127 (12%)	756 (14%)	1,883 (13%)		1,087 (13%)	1,513 (16%)	2,600 (15%)	
	Married	5,182 (55%)	2,315 (43%)	7,497 (51%)		3,465 (42%)	3,442 (36%)	6,907 (39%)	
	Separated/divorced/widowed	992 (10%)	553 (10%)	1,545 (10%)		1,488 (18%)	1,474 (16%)	2,962 (17%)	
	Civil partnership	621 (7%)	359 (7%)	980 (7%)		597 (7%)	705 (7%)	1,302 (7%)	
**Dependent minors**	None	7,074 (74%)	3,497 (66%)	10,571 (71%)	< 0.001	5,58 (67%)	5,875 (62%)	11,455 (65%)	< 0.001
	One	1,141 (12%)	888 (17%)	2,029 (14%)		1,318 (16%)	1,695 (18%)	3,013 (17%)	
	Tw or more	1,284 (14%)	938 (18%)	2,222 (15%)		1,413 (17%)	1,878 (20%)	3,291 (19%)	
**Comorbidities**	None	6,596 (69%)	3,484 (65%)	10,08 (68%)	< 0.001	6,566 (79%)	7,068 (75%)	13,634 (77%)	< 0.001
	One	2,087 (22%)	1,205 (23%)	3,292 (22%)		1,333 (16%)	1,700 (18%)	3,033 (17%)	
	Tw or more	816 (9%)	634 (12%)	1,450 (10%)		412 (5%)	680 (7%)	1,092 (6%)	
**BMI**^1^	Normal weight ([18–25[ kg/m^2^)	3,825 (40%)	2,397 (45%)	6,222 (42%)	< 0.001	4,378 (53%)	4,932 (52%)	9,310 (52%)	< 0.001
	Underweight (< 18 kg/m^2^)	119 (1%)	134 (3%)	253 (2%)		437 (5%)	593 (6%)	1,030 (6%)	
	Overweight ([25–30[ kg/m^2^)	3,857 (41%)	1,839 (35%)	5,696 (38%)		2,215 (27%)	2,266 (24%)	4,481 (25%)	
	Obesity (> 30 kg/m^2^)	1,698 (18%)	953 (18%)	2,651 (18%)		1,281 (15%)	1,657 (18%)	2,938 (17%)	
**SARS-CoV-2 infection**	No infection	8,744 (92%)	4,396 (83%)	13,140 (89%)	< 0.001	7,653 (92%)	8,097 (86%)	15,750 (89%)	< 0.001
	Diagnosed by test or medical examination	197 (2%)	299 (6%)	496 (3%)		239 (3%)	503 (5%)	742 (4%)	
	Suspected	558 (6%)	628 (12%)	1,186 (8%)		419 (5%)	848 (9%)	1,267 (7%)	
**Data collection waves**	Before the "lockdown being lifted" announcement	1,388 (15%)	808 (15%)	2,196 (15%)	0.169	1,112 (13%)	1,354 (14%)	2,466 (14%)	< 0.001
	After the "lockdown being lifted" announcement	5,350 (56%)	3,043 (57%)	8,393 (57%)		4,694 (56%)	5,570 (59%)	10,264 (58%)	
	During the "lockdown lifted" period	2,761 (29%)	1,472 (28%)	4,233 (29%)		2,505 (30%)	2,524 (27%)	5,029 (28%)	
**Frequency of social contacts**	None	5,173 (54%)	2,486 (47%)	7,659 (52%)	< 0.001	4,523 (54%)	4,995 (53%)	9,518 (54%)	0.182
	Low (< 2)	1,835 (19%)	1,217 (23%)	3,052 (21%)		1,615 (19%)	1,926 (20%)	3,541 (20%)	
	Medium ([3, 4])	1,254 (13%)	826 (16%)	2,080 (14%)		1,045 (13%)	1,234 (13%)	2,279 (13%)	
	High (5–200]	1,237 (13%)	794 (15%)	2,031 (14%)		1,128 (14%)	1,293 (14%)	2,421 (14%)	
**Occupational status during lockdown**	At home	7,596 (80%)	4,153 (78%)	11,749 (79%)	0.005	6,804 (82%)	7,834 (83%)	14,638 (82%)	0.067
	Out of home	1,903 (20%)	1,170 (22%)	3,073 (21%)		1,507 (18%)	1,614 (17%)	3,121 (18%)	
**Perceived severity of the pandemic (mean [p25-p75])**		7.72 [7; 9]	7.85 [7; 9]	7.77 [7; 9]	< 0.001	8.00 [7; 9]	8.26 [7; 10]	8.14 [7; 10]	< 0.001
**Total**		9,499 (53%)	5,323 (36%)	14,822 (45%)		8,311 (47%)	9,448 (64%)	17,759 (55%)	

^1^*BMI* Body mass index

The multivariate analyses between education and anxiety-depressive score for men and women are presented in Table 3. Among men, those with a vocational certificate/qualification and those with no diploma had a greater risk of having a high anxiety-depressive score compared to those with a university degree, independent of age and population density (M1: ${\beta }_{Vocational certificate/qualification}$=0.16 [0.04; 0.27];${\beta }_{No diploma}$=0.75 [0.43; 1.07]). Among women, there was a graded association between education level and the risk of having a high anxiety-depressive score: as education level decreased, the anxiety-depressive score increased (M1: ${\beta }_{Baccalaureate}$=0.37 [0.25; 0.49]; ${\beta }_{Vocational certificate/qualification}$=0.41 [0.28; 0.54]; ${\beta }_{No diploma}$=0.80 [0.49; 1.12]). For both men and women, the associations were partly explained by economic and health models, while social context marginally affected this association (M1A; M1B; M1C). Factors related to COVID-19 also partly explained the association between education and mental health, with a stronger contribution observed among women compared to men (M2). When all potential confounders and intermediate variables were included in the model, the absence of diploma remained associated with the risk of presenting a high anxiety-depressive score for men (M3: ${\beta }_{No diploma}$=0.33 [0.02; 0.65]) while for women, all categories of education level remained associated with anxiety-depressive score (M3: ${\beta }_{Baccalaureate}$=0.19 [0.07; 0.31]; ${\beta }_{Vocational certificate/qualification}$=0.17 [0.03; 0.30];${\beta }_{No diploma}$=0.39 [0.07; 0.71]).

**Table 3:** Multivariate linear regressions between education level and anxiety-depressive score adjusted for the different groups of intermediate variables, for men and women (*n* = 32,581)

		***M1***		***M1A***		***M1B***		***M1C***		***M2***		***M3 Full model***	
				**Economic factors**		**Social factors**		**Health factors**		**M1 + Economic factors + Social factors + Health factors**		**M2 + Covid-19 factors**	
	**Education level**	β[CI-95%]	*p*-value	β[CI-95%]	*p*-value	β[CI-95%]	*p*-value	β[CI-95%]	*p*-value	β[CI-95%]	*p*-value	β[CI-95%]	*p*-value
**Men**	University degree	0		0		0		0		0		0	
	Baccalaureate	-0.03	0.67	-0.08	0.23	-0.03	0.58	-0.05	0.4	-0.10	0.12	-0.11	0.07
		[-0.15; 0.09]		[-0.20; 0.05]		[-0.15; 0.09]		[-0.17; 0.07]		[-0.22; 0.03]		[-0.23; 0.01]	
	Vocational certificate/qualification	0.16	0.01	0.09	0.13	0.15	0.01	0.08	0.19	0.01	0.81	-0.06	0.33
		[0.04; 0.27]		[-0.03; 0.22]		[0.03; 0.26]		[-0.04; 0.19]		[-0.11; 0.14]		[-0.18; 0.06]	
	No diploma	0.75	< 0.001	0.64	< 0.001	0.69	< 0.001	0.57	< 0.001	0.42	0.01	0.33	0.04
		[0.43; 1.07]		[0.31; 0.96]		[0.37; 1.01]		[0.25; 0.89]		[0.10; 0.75]		[0.02; 0.65]	
**Women**	University degree	0		0		0		0		0		0	
	Baccalaureate	0.37	< 0.001	0.26	< 0.001	0.36	< 0.001	0.34	< 0.001	0.24	< 0.001	0.19	< 0.001
		[0.25; 0.49]		[0.14; 0.39]		[0.24; 0.48]		[0.22; 0.46]		[0.11; 0.36]		[0.07; 0.31]	
	Vocational certificate/qualification	0.41	< 0.001	0.29	< 0.001	0.40	< 0.001	0.35	< 0.001	0.25	< 0.001	0.17	0.01
		[0.28; 0.54]		[0.16; 0.43]		[0.27; 0.53]		[0.23; 0.48]		[0.11; 0.38]		[0.03; 0.30]	
	No diploma	0.80	< 0.001	0.64	< 0.001	0.77	< 0.001	0.69	< 0.001	0.53	0.001	0.39	0.02
		[0.49; 1.12]		[0.32; 0.97]		[0.45; 1.09]		[0.37; 1.00]		[0.20; 0.85]		[0.07; 0.71]	

• Model 1: Anxiety-depressive score ~ Education level + confounders (age + population density)

∘ Model 1A: Model 1 + Economic factors (occupation + number of people per room living in the accommodation)

∘ Model 1B: Model 1 + Social factors (marital status + dependent minors)

∘ Model 1C: Model 1 + Health factors (comorbidities + BMI)

• Model 2: Model 1 + Economic factors + Social factors + Health factors

• Model 3: Full model: Model 2 + COVID-19 factors (infection status + response period + social contacts + perceived epidemic severity score)

## Discussion

In France, during the COVID-19 pandemic, individuals with a lower level of education had a higher risk of reporting anxiety-depressive symptoms. For both men and women, the association between education and the risk of reporting feelings of depression and anxiety was partly affected by economic and health factors. COVID-19 factors also partly explained the association between education and mental health, with a stronger contribution observed among women compared to men. However, in the fully-adjusted model, the association persisted only for men with no diploma while for women, the whole educational gradient was still observed for the risk of anxiety-depressive state.

Different potential factors have been explored in this study and some of them modified the association between education and anxiety-depressive score among both sexes. Economic factors, which included occupation and overcrowding, affected the relationship between education and anxiety-depressive score. Individuals with low levels of education may be more likely to suffer from chronic economic hardship stress due to their difficulty in accessing a favorable labor market [19] impacting their mental health. We found that the social factors, including marital status and the presence of dependent minors in the household, did not modify the relationship between education and anxiety-depressive score, in neither men nor women. This finding was surprising particularly in women since although the gap is narrowing, women are still more often involved in housework and childcare than men [34], and this resulted in an excessive burden for women during the pandemic [35, 36]. Indeed, in the context of COVID-19 pandemic, mothers were most often involved in home schooling and childcare activities, to the detriment of their work [37, 38]. One possible explanation of our result is that the included variables related to the social factors may affect women in a similar way across all educational categories*.* This may also be related to the nature of the variables used (marital status and dependent minors). The inclusion of other social dimensions such as social support or social network could have produced different results. Health factors, which encompassed comorbidities and BMI, affected the association between education and anxiety-depressive score in both men and women. This is consistent with previous studies that documented associations between lower levels of education and a higher prevalence of obesity [39, 40] as well as associations between lower socio-economic position and increased risk of having comorbidities [41, 42], and BMI and comorbidities are associated with a higher risk of severe forms of mental health disorders [43].

Similarly, the COVID-19 factors affected the relationship between education and anxiety-depressive score. A systemic review of studies conducted across various countries reported that the pandemic increased mental health disorders in the general population [44]. Anxiety generated by the pandemic context may have had an impact on the mental health of individuals, as well as severe COVID-19 illness with prolonged bed rest, which has been found to be associated with long-term mental morbidity in the general population [45]. Furthermore, men and women with a lower level of education may have experienced more severe economic hardship during this period and this may have contributed to a higher level of anxiety-depressive state [2]. An additional contextualization may provide explanations for the role of socio-economic and pandemic-related factors among women. Women make up approximately 70% of the health-care system staff [46] and 60% of sales sector jobs [47]. These roles require exposure to the public, exposure that may not have been suspended during the lockdown, particularly for care positions and essential jobs such as in supermarkets/cashier’s desk. In addition to more exposed and possibly more stressful jobs, women are also more frequently in insecure employment (part-time, fixed-term contracts) in France [48, 49]. Moreover, precarious housing conditions, in particular house crowding, has been associated with detrimental psychological health in women [50].

After accounting for confounders and intermediate variables, we observed a persistent association between education and anxiety-depressive score. The relationship between educational categories (no diploma for men and baccalaureate, vocational certificate/qualification and no diploma for women) and anxiety-depressive score was not fully explained. This persistent association may be explained by earlier or dynamic processes in individuals' lives corresponding to early factors hidden by education [29, 51]. This may be also explained by a low sense of personal control [19] or challenges in health-related knowledge or health literacy [52], affecting the ability to cope with stress and adopt coping behaviours. Differences observed between men and women, with a steeper gradient for women, point to social inequalities in health that may be related to a gender effect [53] previously highlighted in this study population [54]. This issue deserves further investigations.

### Limitations

A number of limitations of this study are important to consider. Firstly, the design is cross-sectional, meaning that the temporal order of variables cannot be accurately ascertained and no causal relationship can be inferred from our results. However, the level of education is mostly determined early in life and is likely to have occurred before the measure of depressive and anxiety symptoms which limits the reverse causality bias. Our study focused on examining the different domains that may contribute to the observed association between education and anxiety-depressive state, which may deserve further investigation to determine their respective potential causal roles using mediation analysis. Secondly, our construction of the anxiety-depressive score is open to discussion as we used self-reported responses to four questions that may not exhaustively measure the mental health of individuals. However, these questions were based on the validated PHQ-4 questionnaire. We could not establish whether there was a certified back-translation process into French for this questionnaire. However, even if this had not occurred, Cronbach's alpha was high (α = 0.86) and ensured a good reliability and internal consistency between the four items measured making up the anxiety-depressive score. Third, we did not include or could not measure all the variables that may impact the association between education and anxiety-depressive state. It is probable that residual confounding factors are present, and that our estimates of the association between education and anxiety-depressive state are overestimated. Fourthly, data used in this work were collected from April 7^th^, nearly three weeks after the lockdown started. Collecting data at the beginning of the lockdown might have led to different results, especially with regard to the level of anxiety-depressive state. The "lockdown being lifted" announcement, which coincided with the earliest data collection waves, could have altered the perceived severity of the pandemic and therefore participants’ anxiety-depressive score. As we do not have data on our study population prior to the COVID-19 period, it is also impossible to say whether the observed associations are related to the pandemic or pre-existed. Finally, our sample may not be representative of the general French population because we conducted our analyses on complete data cases, with a trend to over-represent some individuals compared to the French general population. For example, in our sample, half of the individuals were educated, whereas they were 38% in 2019 [55], and half were over 50, whereas they were 41% in 2023 [56].

## Conclusion

In France, at the end of the first COVID-19 wave, individuals from the general population with a lower level of education had a higher risk of anxiety-depressive state, regardless of their age and area of residence. This association was more pronounced for women, highlighting a process of social inequality in health possibly related to gender. Our findings suggest that these associations may be related to economic factors, individual health conditions or the context of the pandemic. Further investigations using longitudinal data and causal modelling approaches are needed. This study highlights population groups that are potentially vulnerable to mental health problems during a pandemic and should be considered in future public health prevention and intervention actions.

## Data Availability

The dataset(s) supporting the conclusions of this article is an open data set available at: https://www.data.gouv.fr/fr/datasets/datacovid-barometre-covid-19/#/resources under the "Open licence 2.0" granted by the etalab (https://datacovid.org/copyright/).

## Footnotes

1 A public announcement by the President of France, Emmanuel Macron, on 13^th^ April 2020 that the current lockdown measures would be lifted in 4 weeks’ time.
